# Rare Dementia Support Canada: Localizing and mobilizing knowledge to build inclusive supports for families affected by atypical dementias

**DOI:** 10.1002/alz70858_104314

**Published:** 2025-12-26

**Authors:** Mary Pat Sullivan, Sebastian J Crutch, Veronika Williams

**Affiliations:** ^1^ Nipissing University, North Bay, ON, Canada; ^2^ Dementia Research Centre, UCL Queen Square Institute of Neurlogy, London, London, United Kingdom

## Abstract

**Background:**

Literature on how to use theory, evidence and experience to practically develop and implement new interventions for people affected by dementia is scarce. Rare Dementia Support (RDS) is an evidence‐informed support community in the UK for people living with a rare dementia, their families, and dementia care practitioners working in the health and social care sectors. The RDS Impact Study (2019‐24) and the growth of RDS shed new light on the community's characteristics to facilitate a tailored, relational, and continuous model of support. The funding of RDS Canada in 2022 offered an opportunity to localize the model in another jurisdiction. Its implementation was informed by participatory, culturally centred and place‐based solutions.

**Method:**

Following a 2‐year implementation phase and using RDS program theory we conducted an evaluation of RDS Canada's principal activities with its growing membership (∼800 people) and support team. Using multiple data sources and methods, including case studies, an interrogation of administrative data, satisfaction surveys and interviews with staff and advisory circle members (*N* = 17), we explored delivery, reach and implementation successes and challenges across peoples and geography.

**Result:**

People affected by rare dementia living in Canada highlighted a preference for self‐directed support characterized by disease or syndrome, age, and stage, and that provided ‘side by side’ emotional, practical and informational support. Whilst the vitality of the RDS model in Canada was reinforced, as anticipated, the data reinforced the role of pre‐ and post‐diagnostic support that was flexibly available considering family status, culture, language and geography. In a context of shrinking health resources, support that was directed on building member self‐advocacy skills emerged as new and critical contribution to program theory.

**Conclusion:**

The localization and mobilization of knowledge to support people affected by rare dementia providing promising results to continue to develop and expand delivery for diverse populations across diverse geographies in Canada. A newly funded realist evaluation of RDS Canada is now underway to further explore diversity through varied configurations of what supports work for whom, when, and where.

